# The CD8^+^ T Cell-Mediated Immunity Induced by HPV-E6 Uploaded in Engineered Exosomes Is Improved by ISCOMATRIX^TM^ Adjuvant

**DOI:** 10.3390/vaccines4040042

**Published:** 2016-11-09

**Authors:** Francesco Manfredi, Paola di Bonito, Barbara Ridolfi, Simona Anticoli, Claudia Arenaccio, Chiara Chiozzini, Adriana Baz Morelli, Maurizio Federico

**Affiliations:** 1 National AIDS Center, Istituto Superiore di Sanità, Viale Regina Elena, 299, 00161 Rome, Italy; francesco.manfredi@iss.it (F.M.); simona.anticoli@iss.it (S.A.); claudia.arenaccio@iss.it (C.A.); Chiara.chiozzini@iss.it (C.C.); 2 Department of Infectious, Parasitic and Immunomediated Diseases, Istituto Superiore di Sanità, Viale Regina Elena, 299, 00161 Rome, Italy; Chiara.chiozzini@iss.it; 3 Department of Therapeutic Research and Medicine Evaluation, Istituto Superiore di Sanità, Viale Regina Elena, 299, 00161 Rome, Italy; barbara.ridolfi@iss.it; 4 CSL, Ltd., Bio21 Institute, 30 Flemington Road, Melbourne, VIC 3010, Australia; Adriana.Bazmorelli@csl.com.au

**Keywords:** adjuvant, exosomes, Nef, HPV-E6, CD8^+^ T immune response

## Abstract

We recently described the induction of an efficient CD8^+^ T cell-mediated immune response against a tumor-associated antigen (TAA) uploaded in engineered exosomes used as an immunogen delivery tool. This immune response cleared tumor cells inoculated after immunization, and controlled the growth of tumors implanted before immunization. We looked for new protocols aimed at increasing the CD8^+^ T cell specific response to the antigen uploaded in engineered exosomes, assuming that an optimized CD8^+^ T cell immune response would correlate with a more effective depletion of tumor cells in the therapeutic setting. By considering HPV-E6 as a model of TAA, we found that the in vitro co-administration of engineered exosomes and ISCOMATRIX^TM^ adjuvant, i.e., an adjuvant composed of purified ISCOPREP^TM^ saponin, cholesterol, and phospholipids, led to a stronger antigen cross-presentation in both B- lymphoblastoid cell lines ( and monocyte-derived immature dendritic cells compared with that induced by the exosomes alone. Consistently, the co-inoculation in mice of ISCOMATRIX^TM^ adjuvant and engineered exosomes induced a significant increase of TAA-specific CD8^+^ T cells compared to mice immunized with the exosomes alone. This result holds promise for effective usage of exosomes as well as alternative nanovesicles in anti-tumor therapeutic approaches.

## 1. Introduction

Exosomes are vesicles of 50–100 nanometers released by basically all cell types. They are part of the intercellular communication network [1], and are generated by invagination of endosome membranes leading to the formation of intraluminal vesicles which then become part of multivesicular bodies [2].They can traffic to the plasma membrane, thereby releasing their vesicular contents upon membrane fusion. Exosomes have a low intrinsic immunogenic profile, their immunogenicity being related to both amounts and quality of uploaded antigens, and, in some cases, they have been tested in clinical trials [3,4,5]. Exosomes spontaneously uploading tumor antigens, mainly *trans*-membrane proteins like gp100,TRP-1, Her2/neu and carcinoembryonic antigen, induced activation of specific anti-tumor T cell immunity [6,7].

Despite the good tolerance of exosomes as cell-free vaccines, their therapeutic efficacy appeared quite limited, posing the need of new methods to increase their immunogenicity. Attempts to address this issue have been performed by engineering desired antigens to increase their association with the external side of exosome membranes [8,9].

The convergence of exosome and HIV biogenesis at the level of the endosomal sorting complex required for transport (ESCRT) [10] implies the possibility that viral products incorporate in exosomes, as already proven for both Gag [11] and Nef [12,13] HIV-1 proteins. HIV-1 Nef lacks enzymatic activities, yet acts as a scaffold/adaptor element [14]. We identified a Nef mutant (referred to as Nef^mut^) incorporating in HIV-1 virions, virus like particles (VLPs) [15], and exosomes [16] at quite high levels. This Nef mutant is defective for basically all Nef functions, and its efficiency of incorporation in nanovesicles does not change significantly when fused with large foreign proteins [16]. Manipulating Nef^mut^ allows incorporation into exosomes of high amounts of antigens of choice which are protected from external neutralization/degradation factors.

ISCOMATRIX^TM^ (CSL Behring LLC, King of Prussia, PA, USA) adjuvant is an immunostimulating complex formed by ISCOPREP^TM^ (Gronberg Adv. Byra KB, Stockholm, Sweden), saponin extracted from *Quillaia saponaria*, phospholipids, and cholesterol [17]. Its immune adjuvanticity has already been demonstrated, in particular regarding the induction of both CD4^+^ and CD8^+^ T cell immunity directed to soluble protein antigens [18]. We have recently shown that the inoculation in the mice of exosomes engineered to upload high amounts of the TAA HPV-E7 elicits an effective CD8^+^ T cell response [19]. Here, we originally show that ISCOMATRIX^TM^ adjuvant strongly improves both CD8^+^ T-related antigenicity and immunogenicity of proteins delivered by engineered exosomes.

## 2. Materials and Methods

### 2.1. Cell Cultures and Adjuvant

For the experiment, 293T cells were grown in Dulbecco’s Modified Eagle’s Medium (DMEM) plus 10% heat-inactivated Fetal Calf Serum(FCS). The isolation of the Human leukocyte antigen (HLA)-B7 Nef-specific CD8^+^ T cell clone was already described [20]. It recognizes the amino acid sequence TPGPGVRYPL (aa 128–137). Mart-1-specific CD8^+^ T cells recognize the HLA-A.02-restricted AAGIGILTV_27–35_ amino acid sequence. Human monocytes were separated from peripheral blood mononuclear cell (PBMCs) of HLA-A.02 healthy donors using anti-CD14 microbeads (Miltenyi Biotec GmbH, Teterow, Germany), and differentiated to immature iDCs upon 4–5 days of culture in Roswell Park Memorial Institute (RPMI) medium supplemented with 20% FCS, 30 ng/mL granulocyte macrophage colony-stimulating factor (GM-CSF) (AbD Serotec, Bio-Rad Laboratories, s.r.l., Milan Italy), and 500 units/mL IL-4 (R&D Systems, Minneapolis, MN, US). The iDC phenotype was characterized by Fluorescence-activated cell sorting (FACS) analysis for the expression of CD11c and the absence of CD14 cell membrane markers. Both isolation and expansion of the CD8^+^ T cell clone specific for Mart-1 have been previously described [21]. Mouse splenocytes were cultivated in RPMI medium supplemented with 10% FCS. ISCOMATRIX^TM^ adjuvant (CSL Behring LLC, King of Prussia, PA, USA) was prepared as previously described [17], and is composed of ISCOPREP^TM^ saponin, cholesterol, and phospholipids. The undiluted preparation contained 115 ISCO^TM^ Units/mL.

### 2.2. Production, Purification, and Quantification of Exosomes

Exosomes were produced by transiently transfecting 293T cells with vectors expressing Nef^mut^-based fusion proteins. The cells were seeded in the presence of exosome-deprived FCS, and supernatants harvested 48–72 h after transfection. Efficiency of transfection was routinely evaluated by intracellular Fluorescence-activated cell sorting (FACS) analysis as previously reported [22] using the anti-Nef MATG mAb kindly provided by Olivier Schwartz, Pasteur Institute, Paris, France. Exosomes were recovered by differential centrifugations as previously described [23]. The amounts of exosomes were evaluated by measuring the activity of acetylcholinesterase (AchE), i.e., a classical exosome marker [24], through the Amplex Red kit (Molecular Probes, Termo Fischer Scientific, Waltham, MA, USA). The AchE activity was measured as mU/mL, where 1 mU is defined as the amount of enzyme hydrolyzing 1 pmole of acetylcholine to choline and acetate per minute at pH 8.0 at 37 °C.

### 2.3. Fluorescence-Activated Cell Sorting(FACS) Analysis of Bead-Exosome Complexes

Samples were incubated with 5 µL of surfactant-free white aldehyde/sulfate latex beads (Termo Fischer Scientific, Waltham, MA, USA) overnight at room temperature (r.t.) on a rotating plate. For the assays carried out with Nef^mut^/Green Fluorescent protein (GFP) exosomes in the presence of ISCOMATRIX^TM^ adjuvant, beads and exosomes were incubated in the presence of different concentrations of the adjuvant for 1 to 3 h before FACS analysis. For the characterization of different exosome preparations, bead-exosome complexes were labeled with phycoerythrin (PE)-conjugated anti-CD63 mAb (BD Biosciences Milan, Italy) for 1 h at 4 °C. Finally, beads were washed, resuspended in 1× PBS-2% v/v formaldehyde, and FACS analyzed.

### 2.4. Detection of Exosome Cell Internalization

B-lymphoblastoid cell lines (BLCLs) were pre-treated with 100 nM bafilomycin A1 (Sigma-Aldrich, Milan, Italy) for 2 h in the presence or not of ISCOMATRIX^TM^ adjuvant, and then challenged by spinoculation with fluorescent exosomes previously incubated in the presence or not of ISCOMATRIX^TM^ adjuvant. After 2 h of incubation at either 4 or 37 °C in the presence of ISCOMATRIX^TM^ adjuvant and/or bafilomycin A1, cells were treated for 15 min with trypsin, fixed with 2% v/v formaldehyde in 1× PBS, and FACS analyzed.

### 2.5. Western Blot Analysis

The equivalent of 200 µU of exosomes were lysed in PBS, 1% Triton X-100 (Sigma-Aldrich , Milan, Italy) in the presence of anti-proteolytic agents, and then separated in 10% sodium dodecyl sulfate-polyacrylamide gel electrophoresis (SDS-PAGE). Membranes were revealed using sheep anti-Nef antiserum ARP 444, a generous gift of Mark Harris, University of Leeds, Leeds, UK, polyclonal anti-vesicular stomatitis virus G glycoprotein (VSV-G) Abs (Immunological Consultant Laboratories, Newberg, OR, USA), and anti-intercellular adhesion molecule (ICAM)-1 mAb 15.2 (Santa Cruz Biotechnology Inc., Heidelberg, Germany).

### 2.6. Cross-Presentation Assay

HLA-B7 B-LCLs were challenged by spinoculation with Nef^mut^–based exosomes in the presence or not of different concentrations of ISCOMATRIX^TM^ adjuvant. Five hours later, the cells were extensively washed and then co-cultivated in triplicate at 1:2 ratio with Class I major histocompatibility complex (MHC)-matched Nef-CD8^+^ T cells in Elispot multiwell plates pre-coated with the D1K mAb against human interferon (IFN)-γ (Mabtech, Nacka Strand Sweden) in RPMI plus 10% of AB human serum (Gibco, Termo Fischer Scientific, Waltham, MA, USA) for 16 h. Thereafter, co-cultures were removed, the Elispot assay was completed, and the spot-forming cells were analyzed and counted using an Elispot reader (Amplimedical Bioline A-EL-VIS GmbH, Turin, Italy). Cross-presentation assays using Nef^mut^/Mart-1 exosomes were performed basically in the same way, except that HLA-A.02 immature dendritic cells were used as antigen presenting cells (APCs), and the above described Mart-1 specific CD8^+^ T cell clone was used as effector cells.

### 2.7. Immunogenicity Assay

All studies with animals here described have been approved by the Ethical Committee of the Istituto Superiore di Sanità, Rome, Italy (protocol No. 555/SA/2012) according to Legislative Decree 116/92, which has implemented in Italy the European Directive 86/609/EEC on laboratory animal protection. Animals used in our research have been housed and treated according to the guidelines inserted in here above mentioned Legislative Decree. Eight week-old female C57 Bl/6 mice (3 per group in two independent experiments) were purchased from Charles River Laboratories Italia, (Calco, Italy) and inoculated s.c. 3 times at 2 week intervals with a total of 100 µL comprising Nef^mut^/E6 exosomes in the presence or not of ISCOMATRIX^TM^ adjuvant. Two weeks after the last inoculation, mice were sacrificed, and splenocytes put in culture in the presence of 5 µg/mL of 8- or 9-mer HPV-E6 peptides already identified to efficiently bind the H-2 K^b^ complex of C57 Bl/6 mice [25], i.e., KLPQLCTEL (aa. 18–26) and YDFAFRDL (aa 50–57). H-2 K^b^ binding HPV E7-specific peptides DLYCYEQL (aa 21–28), and RAHYNIVTF (aa 49–57) [25] were used as control peptides. IFN-γ Elispot assays were performed after overnight incubation in Elispot microwells. All IFN-γ Elispot assays were performed in triplicate conditions using commercially available reagents (Mabtech AB, Nacka Strand Sweden), and spots counted using an Elispot reader.

### 2.8. Detection of Anti-HPV-E6 Abs

Sera from inoculated mice were pooled, and two-fold serial dilutions starting from 1:100 were assayed for the presence of anti-HPV-E6 Abs as previously reported [19] using recombinant HPV-E6 produced as described [26].

### 2.9. Cytotoxic T Lymphocyte (CTL) Assay

CD8^+^ T cells were isolated from splenocytes of exosome-inoculated mice by positive immunomagnetic selection (Miltenyi Biotec., Teterow, Germany). They were put in co-culture with EL-4 cells previously labeled with carboxyfluorescein succinimidyl ester (CFSE, Invitrogen, Termo Fischer Scientific, Waltham, MA, USA) and treated overnight with either E6 or unrelated peptides. The co-cultures were run for 6 h in 200 µL of RPMI 20% FCS in U-bottom 96 well plates at 20:1 effector/target cell ratio. Afterwards, EL-4 cell mortality was scored by FACS analysis soon after addition of 7-AAD at a final concentration of 1 µg/mL.

### 2.10. Statistical Analysis

When appropriate, data are presented as mean + standard deviation (SD). In some instances, the paired Student’s *t*-test was used and confirmed using the non-parametric Wilcoxon rank sum test. *p* < 0.05 was considered significant.

## 3. Results

### 3.1. ISCOMATRIX^TM^ Adjuvant Does Not Affect Both Structure and Cell Entry Efficiency of Exosomes

**Figure 1:**
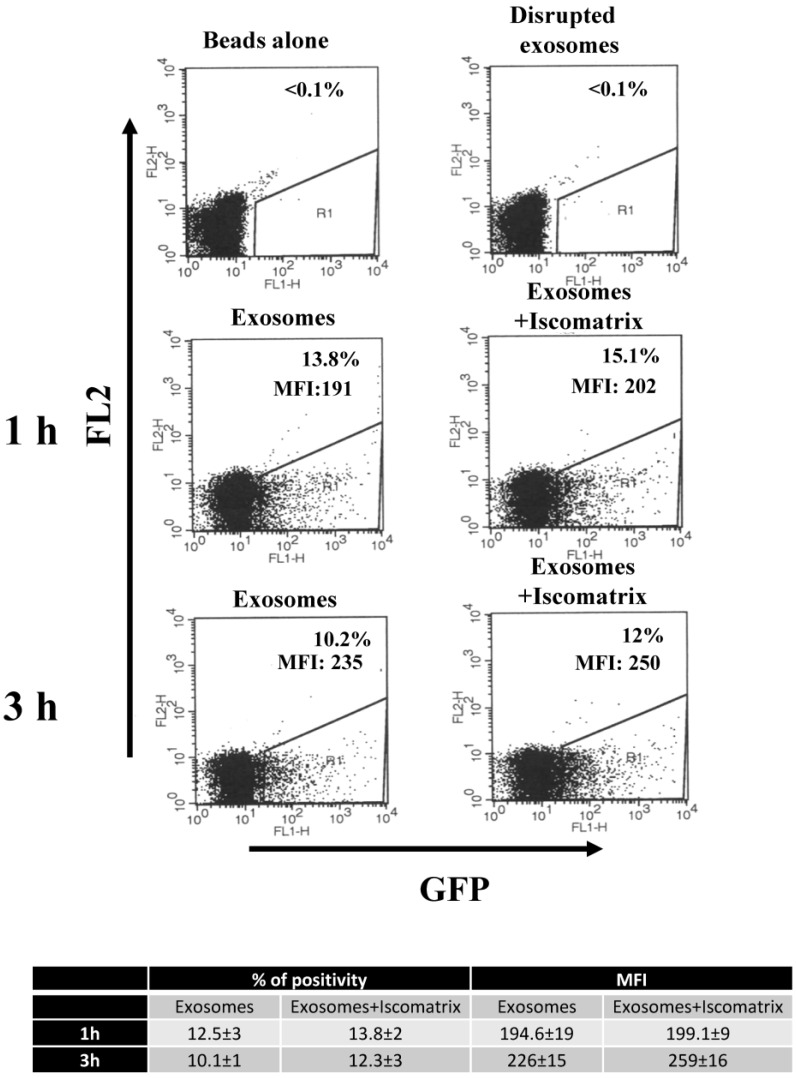
The treatment with ISCOMATRIX^TM^ adjuvant does not affect the exosome-associated fluorescence. Fluorescent exosomes were incubated in the absence or presence of 23 ISCO^TM^ U/mL of the adjuvant at 37 °C with aldehyde sulphate beads in a rotating plate. After 1 and 3 h, samples were washed, and analyzed by FACS. As a control, equivalent amounts of fluorescent exosomes were heated at 95 °C for 10 min before incubation with beads. Results representative of three independent experiments with duplicates are shown. In each plot, both percentages and, where relevant, mean fluorescence intensity (MFI) of fluorescent beads are indicated. At the bottom, mean values ± SD of both percentages and MFI of fluorescent beads from the three experiments are reported. Iscomatrix: ISCOMATRIX^TM^ adjuvant.

Attempting to improve the potency of the antigen-specific CTL response that we previously observed in mice inoculated with Nef^mut^-based exosomes, we looked for adjuvants already proven to increase the CD8^+^ T lymphocyte response, and whose molecular composition was expected to not impact the exosome structure. ISCOMATRIX^TM^ adjuvant appeared as a valuable candidate. However, preliminary experiments aimed at identifying possible structural and/or functional alterations of exosomes upon interaction with ISCOMATRIX^TM^ adjuvant were mandatory. Structural alterations were evaluated using fluorescent exosomes whose GFP contents were measured by FACS analysis after binding to aldheyde-sulphate beads. In this system, we assumed that relevant damages in the exosome structure couple with a loss of the exosome-associated fluorescence. In detail, GFP-labeled exosomes were recovered by transiently transfecting 293T cells with a vector expressing the Nef^mut^/GFP fusion protein as previously described [16]. A total of 1 mU of these exosomes were bound to the beads, and then incubated with either 5.75, 11.5, or 23 ISCO^TM^ U/mL of ISCOMATRIX^TM^ adjuvant for 1 to 3 h. As a control, the same amount of fluorescent exosomes was disrupted by heating at 95 °C for 10 min in water before the incubation with the beads. Figure 1 shows the results obtained with the highest concentration of the adjuvant. Clearly, no differences in the beads-associated fluorescence appeared between control and ISCOMATRIX^TM^ adjuvant -treated exosomes, and similar results were obtained with lower adjuvant concentrations (not shown). This result strongly suggested that the incubation with ISCOMATRIX^TM^ adjuvant does not induce degradation of exosomes.

**Figure 2:**
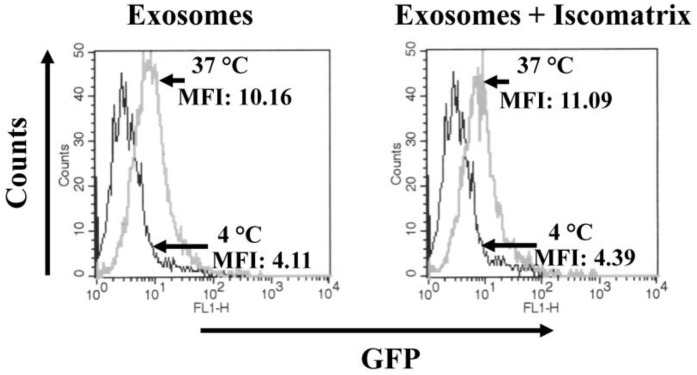
Unchanged cell internalization of exosomes in the presence of ISCOMATRIX^TM^ adjuvant. A total of 2 × 10^5^ B-LCLs were pre-treated for 2 h with bafilomycin A1 in the presence or not of the ISCOMATRIX^TM^ adjuvant, and then challenged with 500 µU of fluorescent exosomes pre-incubated or not with the adjuvant. After spinoculation, the cells were incubated for an additional 2 h at either 4 or 37 °C in the presence of bafilomycin A1. Finally, cells were fixed and FACS analyzed. Fluorescence intensity of cells incubated at 4 °C was similar to that of untreated cells. Shown are the results representative of two independent experiments carried out with duplicates. Mean fluorescence intensity (MFI) of the samples are also indicated. Iscomatrix: ISCOMATRIX^TM^ adjuvant.

Next, we looked at possible influences of ISCOMATRIX^TM^ adjuvant on cell entry efficiency of exosomes. To this end, fluorescent exosomes were incubated for two hours with complete medium either alone or supplemented with 23 ISCO^TM^ U/mL in a total of 50 µL. Meanwhile, cells were pre-treated for 2 h with the adjuvant and/or bafilomycin A1, the latter required to hamper rapid intracellular degradation of incoming exosomes. Afterwards, B-LCLs were challenged with exosomes by spinoculation carried out either at 4 or 37 °C. Cells were then re-plated at the two different temperatures, and after 2 h treated with trypsin, and finally FACS analyzed. As shown in Figure 2, fluorescence levels in B-LCLs did not change significantly when exosomes were pre-treated with ISCOMATRIX^TM^ adjuvant, indicating that the adjuvant does not affect the cell entry efficiency of exosomes.

From this set of experiments, we concluded that both structure and cell entry of exosomes are not influenced by the interaction with ISCOMATRIX^TM^ adjuvant.

### 3.2. ISCOMATRIX^TM^ Adjuvant Increases Cross-Presentation of Antigens Uploaded in Engineered Exosomes

For exogenous antigens, cross-presentation is on the basis of the CD8^+^ T cell immune adaptive response. Thus, we first tested the efficiency of the exosome-ISCOMATRIX^TM^ adjuvant combination in an in vitro cross-presentation assay assuming that the extents of cross-presentation of antigens uploaded in engineered exosomes detectable in vitro predicts the potency of the immune response in vivo. The effects of ISCOMATRIX^TM^ adjuvant on the cross-presentation of antigens associated with engineered exosomes were evaluated in two already established in vitro systems based on challenging B-LCLs and iDCs with exosomes uploading Nef^mut^ alone [16] and fused with Mart-1 (i.e., a human melanoma-associated antigen also known as Melan-A) [27], respectively. We previously reported that, in this system, the antigens delivered by exosomes are cross-presented poorly unless the exosomes associate with a pH-dependent envelope protein (e.g., the G protein from vesicular stomatitis virus) [16].

**Figure 3:**
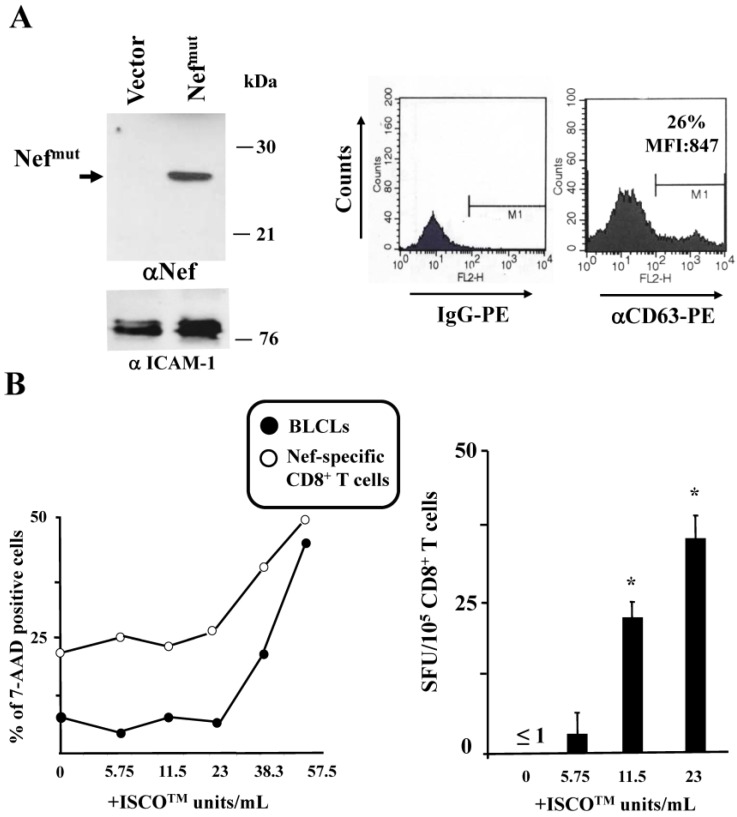
ISCOMATRIX^TM^ adjuvant increases the cross-presentation of antigens delivered by engineered exosomes in B-lymphoblastoid cell lines (LCLs). (**A**) molecular characterization of exosome preparations uploading Nef^mut^. On the left is the Western blot analysis of 200 µU of exosomes uploading Nef^mut^. As a control, equivalent amounts of exosomes from cells transfected with the empty vector were loaded. Arrow signs indicate the relevant protein product. Exosome preparations were also probed for the presence of Intercellular adhesion molecule (ICAM)-1, i.e., an exosome marker. Molecular markers are given in kilodaltons (kDa). On the right, fluorescence-activated cell sorting (FACS) analysis for the presence of CD63 in exosome membrane uploading Nef^mu^ is shown. M1 marks the range of fluorescence positivity as determined by the analysis of equivalent amounts of exosomes after incubation with isotype-specific IgGs (**left** histogram). Both percentages and MFI of fluorescent beads are indicated. Results shown in both panels are representative of three assays carried out on two exosome preparations; (**B**) on the left is cell viability of both B-LCLs and Nef-specific CD8^+^ T cells treated for 24 h with the indicated concentrations of ISCOMATRIX^TM^ adjuvant as evaluated by FACS analysis after 7-AAD labeling. Shown are mean values from two independent experiments with duplicates. On the right, data from cross-presentation analysis of Nef^mut^ delivered by exosomes in B-LCLs are presented. A total of 10^5^ cells were challenged with 100 µU of exosomes and then cultivated for 5 h in the presence of the indicated doses of ISCOMATRIX^TM^ adjuvant. Thereafter, the cells were put in co-culture overnight with a Nef-specific, HLA-B7 restricted CD8^+^ T cell line in IFN-γ Elispot microwells. Shown are the mean + SD number of SFU/10^5^ cells calculated from five independent experiments. * *p* < 0.05.

Exosomes uploading Nef^mut^ were produced by transient transfection in 293T cells as previously described [16], and characterized for both Nef^mut^ incorporation and CD63 membrane expression (Figure 3A). HLA-B7 B-LCLs were challenged with Nef^mut^-based exosomes alone or in combination with either 5.75, 11.5, or 23 ISCO^TM^ U/mL of ISCOMATRIX^TM^ adjuvant. Notably, no effects on growth/viability of both B-LCLs and Nef-specific CD8^+^ T lymphocytes were assessed after 24 h of cultivation with these doses of the adjuvant (Figure 3B, left). Exosome challenge was carried out by spinoculating cells in a total of 50 µL, followed by a 5 h incubation in the presence or not of the indicated doses of ISCOMATRIX^TM^ adjuvant (Figure 3B, right), and thereafter co-cultivated for 24 h with HLA-matched Nef-specific CD8^+^ T lymphocytes in IFN-γ Elispot microwells. As shown in Figure 3B, the presence of at least 11.5 ISCO^TM^ U/mL of the adjuvant significantly increased the cross-presentation of Nef^mut^ delivered by exosomes.

**Figure 4:**
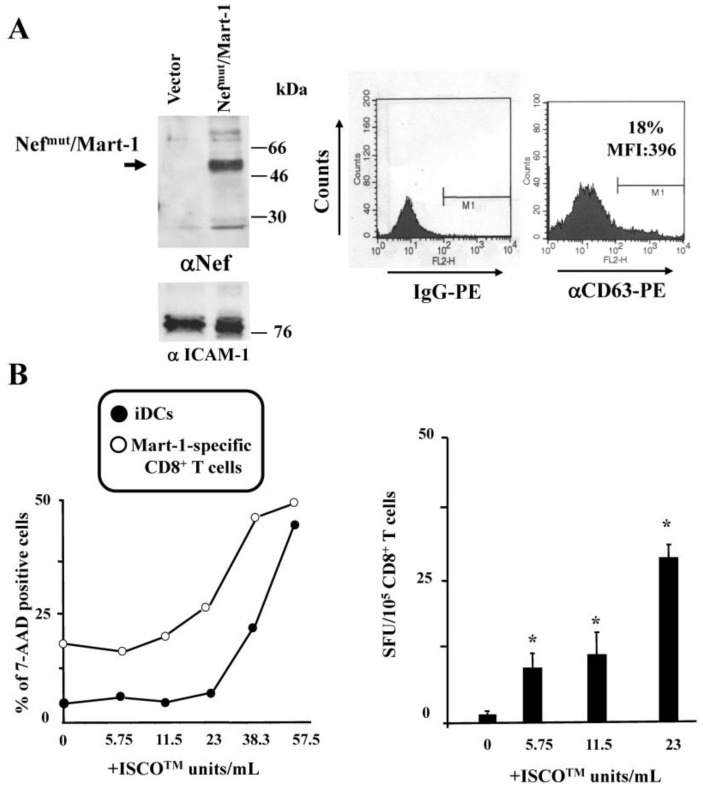
ISCOMATRIX^TM^ adjuvant increases the cross-presentation of antigens delivered by engineered exosomes in iDCs. (**A**) molecular characterization of exosome preparations uploading Nef^mu^/Mart-1. On the left is the Western blot analysis of 200 µU of exosomes associating with Nef^mut^. As a control, equivalent amounts of exosomes from cells transfected with the empty vector were loaded. Arrow signs indicate the relevant protein product. Exosome preparations were also probed for the presence of ICAM-1. Molecular markers are given in kDa. On the right, the FACS analysis for presence of CD63 in exosome membrane is shown. M1 marks the range of fluorescence positivity as determined by the analysis of equivalent amounts of exosomes after incubation with isotype-specific IgGs (**left** histogram). Both percentages and MFI of fluorescent beads are indicated. Results shown in both panels are representative of two assays carried out on two exosome preparations; (**B**) on the left is cell viability of both iDCs and Mart-1-specific CD8^+^ T cells treated for 24 h with the indicated concentrations of ISCOMATRIX^TM^ adjuvant as evaluated by FACS analysis after 7-AAD labeling. Mean values from two independent experiments with duplicates are shown. On the right, results from cross-presentation analysis of Mart-1delivered by Nef^mut^/Mart-1 exosomes in HLA-A.02 iDCs are presented. A total of 10^5^ cells were challenged with 100 µU of the exosomes and then cultivated for 5 h in the presence of the indicated doses of ISCOMATRIX^TM^ adjuvant. Thereafter, the cells were put in co-culture overnight with a Mart-1-specific, HLA-A.02 restricted CD8^+^ T cell line in IFN-γ Elispot microwells. The mean + SD number of SFU/10^5^ cells calculated from three independent experiments are shown. * *p* < 0.05.

The cross-presentation assay was reproduced using monocyte-derived iDCs and exosomes uploading Nef^mut^/Mart-1, whose molecular characterization is shown in Figure 4A. When these exosomes were used to challenge HLA A.02 iDCs, whose viability appeared not influenced up until 23 ISCO^TM^ U/mL (Figure 4B, left), the increase of Mart-1 cross-presentation appeared from 5.75 ISCO^TM^ U/mL of ISCOMATRIX^TM^ adjuvant (Figure 4B, right). According to what was already reported [28], ISCOMATRIX^TM^ adjuvant had no detectable effects on activation/maturation of in vitro differentiated iDCs, independently from the exosome treatment (not shown).

Of note, incubation of both B-LCLs and iDCs with the adjuvant in the presence of exosomes from cells transfected with empty vector did not induce activation of the CD8^+^ T cells (not shown).

These results indicated together that the treatment with ISCOMATRIX^TM^ adjuvant significantly increases the cross-presentation of antigens uploaded in Nef^mut^-based engineered exosomes.

### 3.3. ISCOMATRIX^TM^ Adjuvant and Exosome Co-Administration in Mice Increases the Pool of CD8^+^ T Lymphocytes Specific for the Antigen Uploaded in Engineered Exosomes

**Figure 5:**
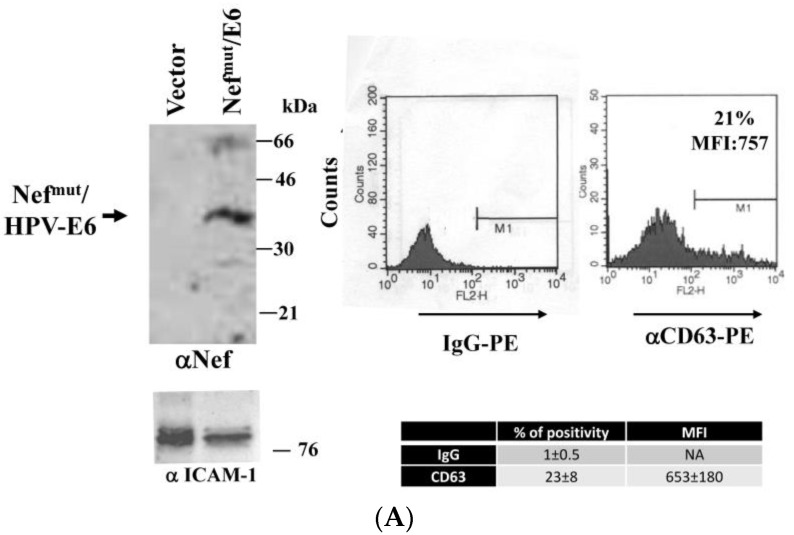

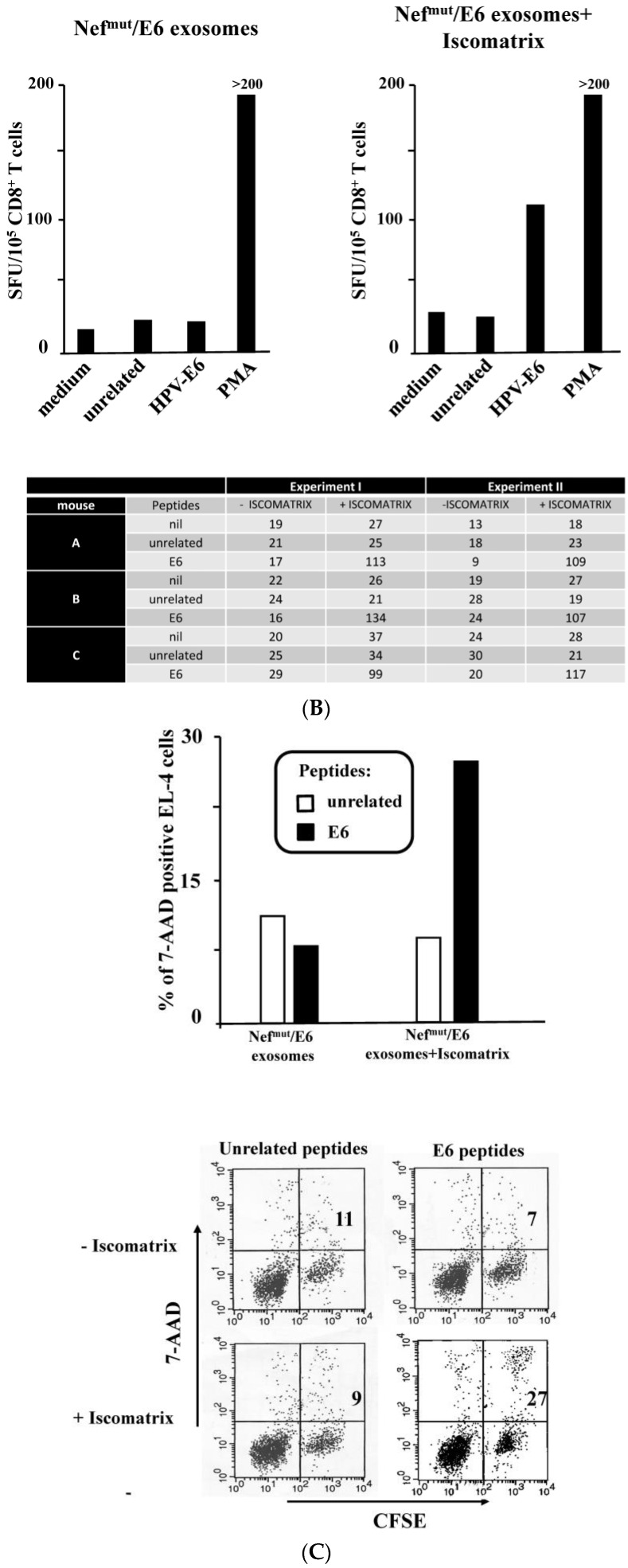

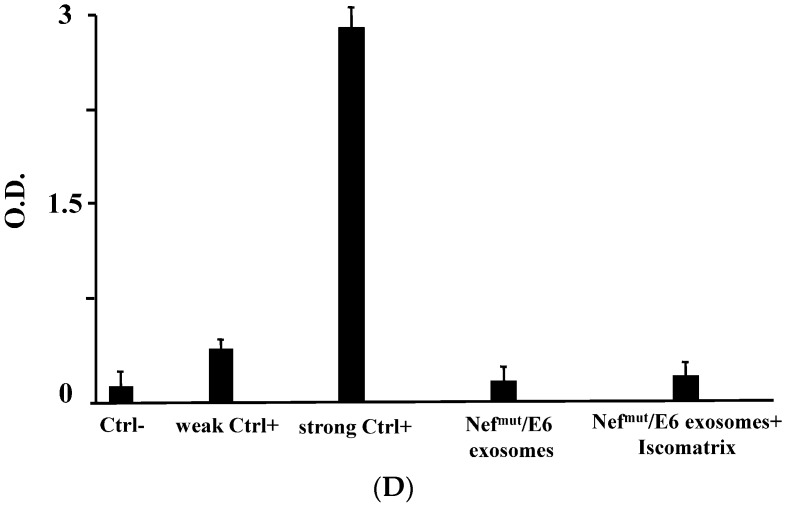
The co-inoculation in mice of ISCOMATRIX^TM^ adjuvant and engineered exosomes increases the number of HPV-E6 specific CD8^+^ T lymphocytes. (**A**) molecular characterization of exosome preparations uploading Nef^mu^/HPV-E6. On the left is the Western blot analysis of 200 µU of exosomes associating Nef^mut^/E6. As a control, equivalent amounts of exosomes from cells transfected with the empty vector were loaded. Arrow signs indicate the relevant protein product. Exosome preparations were also probed for the presence of ICAM-1. Molecular markers are given in kDa. On the right, the FACS analysis for the presence of CD63 in exosome membrane is shown. M1 marks the range of fluorescence positivity as determined by the analysis of equivalent amounts of exosomes after incubation with isotype-specific IgGs (**left** histogram). Results shown in both panels are representative of five assays carried out on three exosome preparations. At the bottom right, mean values ±SD of both percentages of positivity and MFI from the five assays are also reported; (**B**) CD8^+^ T cell immune response in mice inoculated with Nef^mut^/E6 exosomes in the presence or not of ISCOMATRIX^TM^ adjuvant. C57 Bl/6 mice (three per group) were inoculated s.c. at the lower right flank three times with exosomes uploading Nef^mut^/E6 in the presence or not of the adjuvant. Two weeks after the last inoculation, splenocytes were isolated and 10^5^ cells were incubated overnight with or without 5 µg/mL of either unrelated or HPV-E6-specific peptides in IFN-γ Elispot microwells in triplicate conditions. As a control, untreated cells were incubated with 10 ng/mL of phorbol-12-myristate-13-acetate (PMA) and 500 ng/mL of ionomycin. Afterwards, cell activation extents were evaluated by IFN-γ Elispot assay carried out in triplicate with 10^5^ cells/well. Cultures of splenocytes from each inoculated mouse were carried out separately. The mean of SFU/10^5^ cells calculated on the basis of data reported at the bottom which were obtained in two independent immunization experiments are shown. Iscomatrix: ISCOMATRIX^TM^ adjuvant; (**C**) Cytotoxic T lymphocyte (CTL) assay carried out with CD8^+^ T cells isolated from splenocytes of mice inoculated with exosomes uploading Nef^mut^/E6 in the presence or not of ISCOMATRIX^TM^ adjuvant. CD8^+^ T lymphocytes from pooled splenocyte cultures were co-cultivated for 6 h at 20:1 effector/target cell ratio with EL (European lymphoblast)-4 cells previously labeled with Carboxyfluorescein succinimidyl ester (CFSE), and treated with either unrelated or E6 peptides for 16 h. Finally, the EL-4 cell mortality levels were scored by FACS analysis upon 7-AAD labeling. At the **top**, are the results obtained using pooled splenocytes from a representative of two independent immunization experiments. At the **bottom**, the dot-plot FACS analysis of the co-cultures is reported. Cells were gated on the basis of their apparently unaffected morphology, and the percentages of double-positive over the total of CFSE-labelled cells are reported; (**D**) anti-E6 antibody detection in plasma from mice inoculated with the Nef^mut^/E6 exosomes in the presence or not of ISCOMATRIX^TM^ adjuvant. Plasma were tested in an in-house Elisa assay upon 1:10 dilution. As internal standards, 1:10 diluted plasma from mock-inoculated mice was used as negative control (Ctrl−), whereas both 1:1000 (weak Ctrl+) and 1:100 (strong Ctrl+) dilutions of plasma from mice injected with 10 µg of recombinant E6 protein plus adjuvant were used as positive controls. Shown are the mean absorbance values +SD of triplicates of plasma samples from each inoculated mouse.

The aim of the present study was the identification of a method to improve the CD8^+^ T cell immunity induced by the inoculation of Nef^mut^-based exosomes. Hence, we next were interested in assessing whether the ISCOMATRIX^TM^ adjuvant-dependent improvement of cross-presentation activity that we observed in vitro was associated with increased immunogenicity in animals. To this aim, exosomes engineered with the Nef^mut^/HPV-E6 fusion protein were purified from the supernatants of 293T transfected cells and characterized in terms of contents of both the fusion product and CD63 tetraspanin (Figure 5A). C57Bl/6 mice were inoculated subcutaneously (s.c.) three times with two week intervals with 2.1 mU of Nef^mut^/E6 exosomes in the presence or not of 3.8 ISCO^TM^ Units of ISCOMATRIX^TM^ adjuvant, i.e., the highest dose well tolerated by s.c. injected mice. Two weeks after the last immunization, mice were sacrificed, and splenocytes assayed for the presence of HPV-E6-specific CD8^+^ T lymphocytes. By IFN-γ Elispot assay, we detected HPV-E6-specific CD8^+^ T lymphocytes in splenocytes from mice inoculated with Nef^mut^/E6 exosomes plus ISCOMATRIX^TM^ adjuvant, but not from mice inoculated with the exosomes alone (Figure 5B). The latter result was consistent with the previously reported evidence that the CD8^+^ T cell response against HPV-E7 delivered by engineered exosomes was detectable only after a 5-day culture of splenocytes in the presence of specific peptides [19]. The increased number of E6-specific CD8^+^ T cells within splenocytes from mice co-inoculated with ISCOMATRIX^TM^ adjuvant correlated with the appearance of a E6-specific CTL activity, as shown by the results we obtained through a CTL assay based on the co-culture with syngeneic EL-4 cells pre-treated with E6 peptides (Figure 5C). Of note, no anti-HPV-E6 antibodies were found in sera from mice inoculated with Nef^mut^/E6 incorporating exosomes whatever the co-inoculation of ISCOMATRIX^TM^ adjuvant (Figure 5D).

From these data, we concluded that ISCOMATRIX^TM^ adjuvant is instrumental in increasing the CD8^+^ T cell immune response against exosome-associated antigens.

## 4. Discussion

Nef^mut^-based exosomes represent an original tool for the induction of a Class I MHC-unrestricted CTL immune response against antigens of choice. The basically unvaried efficiency of exosome incorporation of Nef^mut^ when fused at its C-terminus with an heterologous antigen guarantees the great flexibility of this immunogen platform. Considering that the inoculation of Nef^mut^-based exosomes does not induce specific Abs, leading exclusively to antigen-specific CD8^+^ T lymphocyte immunity, adjuvants already characterized for their ability to increase this arm of the adaptive immunity could represent useful tools to strengthen the immunogenicity of Nef^mut^-based exosomes.

Exosomes engineered to upload HPV-E7 fused with the Nef^mut^ exosome-anchoring protein have been recently shown to efficiently elicit a CD8^+^ T-specific adaptive immune response against E7 [19].This immune response blocked the growth of tumor cells implanted after immunization, appearing however only partly efficient in the therapeutic setting. Here, we present a simple method to increase the immunogenicity of the Nef^mut^-based exosomes useful for possible therapeutic applications. The increase of antigen-specific CTL immunity that we documented in splenocytes from ISCOMATRIX™ adjuvant-co-inoculated mice is expected to be associated with increased survival of animals challenged with HPV E6-expressing tumor cells, as we already demonstrated for HPV E7- engineered exosomes [19]. To the best of our knowledge, for the first time, the activity of a saponin-based adjuvant favoring the immunogenicity of a nanovesicle-delivered antigen has been demonstrated.

Cross-presentation in DCs relies on two non-mutually exclusive mechanisms [29]. In the first one, referred to as “cytosolic”, the antigen transits from the endosomal compartment to the cytosol. In the case of endocytosed vesicles, this passage can be greatly favored by pH-dependent envelope fusion proteins. In cytosol, the antigen is degraded by proteasome, and resulting peptides are loaded on Class I MHC upon Transporter associated with antigen processing (TAP)-mediated translocation into endoplasmatic reticulum. The second mechanism, defined as “vacuolar”, is based on the action of endo-lysosomal proteases degrading both vesicles and associated proteins, whose resulting peptides are loaded into Class I MHC recycling at vesicular levels. We assumed that the CD8^+^ T-related immunogenicity that we observed using exosomes produced in the absence of foreign envelope proteins was a consequence of the vacuolar cross-presentation activity in APCs ingesting the exosomes.

We selected an ISCOMATRIX™ adjuvant mainly based on our in-depth understanding of its mechanism of action. ISCOMATRIX™ adjuvant induces integrated responses including antibody and cellular immune responses to various types of soluble antigens [17]. The particulate nature of ISCOMATRIX™ adjuvant (40–50 nm) contributes to some of its properties. ISCOMATRIX™ adjuvant is efficiently endocytosed by APCs where it exerts its immunomodulatory activities. Generation of high frequency antigen-specific CD8^+^ T cell responses is a reproducible feature of ISCOMATRIX™ adjuvant vaccines in both animal models and in humans [28,30,31]. We have already demonstrated that ISCOMATRIX™ adjuvant induces prolonged antigen cross-presentation persisting in vivo up to seven days after priming, which, together with efficient Ag delivery, provides a mechanistic explanation for the strong CD8^+^ T cell responses induced by ISCOMATRIX™ adjuvant vaccines [32]. In the same study, it was also demonstrated that ISCOMATRIX™ adjuvant vaccines are more efficient at inducing CTL responses in vivo than other adjuvants such as aluminum hydroxide, incomplete Freund’s adjuvant, CpG, lipopolysaccharides (LPS) or Pam3Cys. Wilson and colleagues further reported that cells (at both draining lymph nodes and injection site) that directly encounter/take up ISCOMATRIX™ adjuvant likely undergo metabolic cell stress, which triggers multiple “danger” signaling pathways [33]. All of these effects could represent an advantage compared to using other adjuvants which either trigger only one particular pathway, or having undefined mechanism of action.

It was already shown that ISCOMATRIX^TM^ adjuvant significantly increases the cross-presentation of soluble antigens [17]. Although the exact underlying mechanisms remains to be fully elucidated, it has been reported that in vivo it: (i) induces DC activation; (ii) facilitates antigen cross-presentation in a specific subset of DCs, i.e., the CD8α^+^; and (iii) generates a pro-inflammatory milieu in the inoculation site, with increased production of both IL-1β and IL-6 [29]. An inflammatory milieu leading to activation of professional APCs is expected to favor adaptive immune responses. However, whether and how inflammatory factors are relevant for cross-presentation of exosome-associated antigens remains to be established.

ISCOMATRIX^TM^ adjuvant has also been proven to boost the antibody response against soluble antigens [17]. In our hands, consistently with what previously reported for HPV E7-uploaded engineered exosomes [19], no antibody response against HPV-E6 has been detected in mice challenged with exosomes incorporating Nef^mut^/E6 also in the presence of ISCOMATRIX^TM^ adjuvant. This evidence was strongly suggestive of a basically exclusive presentation of HPV-E6 peptides in Class I MHC also in the presence of the adjuvant.

Anti-tumor experimental vaccine strategies described so far are based on the use of either peptides, recombinant proteins, DNA, viral vectors, or VLPs. Some intrinsic limitations reduce the possibility of effective transfer to clinics for many of these approaches. The use of the engineered exosomes in anti-tumor vaccine strategies described here presents a number of advantages including: (i) a quite low basal immunogenicity; (ii) the incorporation of the whole tumor-associated antigen, which implies a Class I MHC-unrestricted use; (iii) the lack of genetic material, and a minimal presence of non-human antigens; (iv) a manufacturing simpler than that required for viral vectors and VLPs. The promising results that we obtained justify further investigations on both immunogenicity and efficacy of exosomes engineered with additional antigens of therapeutic significance.

## 5. Conclusions

Overall, our results indicate that the molecular composition of ISCOMATRIX^TM^ adjuvant is perfectly compatible with the structural integrity of exosomal nanovesicles. ISCOMATRIX^TM^ adjuvant increases cross-presentation of exosome-associated antigens in vitro, and consistently improves the CD8^+^ T cell response against the foreign antigen incorporated in engineered exosomes. Hence, besides soluble antigens, ISCOMATRIX^TM^ adjuvant is also useful for increasing the immunogenicity of antigens incorporated in nanovesicles. This result could be of utility for both current and future immunotherapies based on nanovesicle-associated antigens.
